# Ethanol lock prophylaxis in long-stay central venous catheters in children with severe intestinal dysfunction: a report of six cases

**DOI:** 10.1590/1677-5449.202102211

**Published:** 2022-07-06

**Authors:** Mário Cícero Falcão, Gabriela Ibrahim Martins de Castro, Juliana Valeria de Souza Framil, Juliana Zoboli Del Bigio, Ana Cristina Aoun Tannuri

**Affiliations:** 1 Universidade de São Paulo – USP, Instituto da Criança e do Adolescente, Faculdade de Medicina, Hospital das Clínicas, São Paulo, SP, Brasil.

**Keywords:** short bowel syndrome, parenteral nutrition, total, catheter-related infections, ethanol

## Abstract

The aim of this study was to report on use of ethanol lock in long-term catheters in newborns with severe intestinal dysfunction, dependent on total and prolonged parenteral nutrition, in a Neonatal Intensive Care Center (tertiary level), between 2015 and 2020. Six infants (0.65%) out of the 914 admitted during the period met the inclusion criteria. The median age at catheter placement was 121.5 days. Two Powerpicc (PICC Power Sinergy™, São Paulo), one Groshong (Groshong™ Central Venous Catheter BD, São Paulo), and three silicone catheters were used, all tunneled, and the median dwell duration was 182.5 days. Four patients had at least one episode of infection related to the central venous catheter, and Gram-positive, Gram-negative, and fungal agents were isolated. The median length of hospital stay was 555 days and mortality was 33.3%. The ethanol lock did not cause any side effects and was relatively effective in preventing infections related to the central venous catheter.

## INTRODUCTION

Loss of intestinal function can be the result of absent intestine, absorption disorders, motility disorders, or a combination of these. One classic example of this association is short bowel syndrome, in which there is insufficient intestine, primarily small intestine, to enable adequate digestion and absorption of nutrients to sustain life.^1^^,^ 2

In newborn infants, the most common cause of short bowel syndrome resulting from surgical resection is necrotizing enterocolitis (35 to 50% of cases). Other possibilities include defects of the abdominal wall (gastroschisis and omphalocele), malrotation, volvulus, and multiple intestinal atresias.^3^^,^ 4 Management of intestinal dysfunction is the same regardless of cause and includes providing the conditions for intestinal adaptation and recovery to establish autonomy, i.e., independence from parenteral nutrition.^5^

Septic complications related to central venous catheter infections are the main causes of mortality, with rates varying from 20 to 40%, depending on prevention of complications.^6^

A primary bloodstream infection is considered to be related to a central venous catheter when it is documented that there is a greater pathogen load at the catheter than in peripheral blood, whether by quantitative analysis of catheter tip cultures (identifying the same pathogen as in peripheral blood, with more than 15 CFU/plate in a semi-quantitative catheter tip culture or more than 100 CFU/mL in a quantitative culture), by the difference in concentration between central and peripheral blood cultures (growth at least three times greater in a quantitative culture of blood from the central venous catheter than in a peripheral blood culture), or by the difference in positivity time between central and peripheral blood cultures (faster growth in the central venous catheter blood culture, with a difference in positivity time greater than 120 minutes).^7^

Measures are therefore being implemented to reduce the risk of central venous catheter infections, such as emphasizing correct hand hygiene practices for catheter insertion, adoption of bundles of measure that cover precautions for catheter maintenance, such as improved dressing surveillance or early removal of devices, when possible, and precautions related to handling of catheters, especially during bathing, blood draws, and administration of medications.^7^

In addition to these basic measures, substances can be employed to prevent build up of biofilm in the catheter, using a technique known as a prophylactic lock.^8^ The main methods for avoiding formation of biofilms are locks employing antibiotics, fibrinolytics, and ethanol.^1^ A systematic review published in 2017 and including pediatric and adult patients with cancer showed that the antimicrobial lock technique can serve as an adjuvant to prevention of bloodstream infections in patients with central venous catheters. However, better quality evidence is needed to support specific recommendations.^9^

## CASE DESCRIPTIONS

The inclusion criteria for clinical cases were as follows: presence of severe intestinal insufficiency, prolonged fasting, total parenteral nutrition infused via a silicone central venous catheter implanted with the tunneling technique, and use of prophylactic ethanol lock for 4 hours. From 2015 to 2020, a total of 914 newborn infants were admitted to the unit, six of whom fulfilled the criteria listed above.

Tables 1, 2, and 3 summarize the case descriptions, showing that 66.6% had short bowel syndrome and median catheter dwell duration was 182.5 days. No side effects of the prophylactic ethanol lock were observed and 83.3% of the patients exhibited at least one episode of central venous catheter-related infection, from whom Gram-positive, Gram-negative, and fungal agents were isolated. Median length of hospital stay was 555 days, and mortality was 33.3%, although no deaths occurred while infants were in the unit. All three deaths were caused by sepsis with multiple organ failure.

**Table 1:** Gestational age (weeks), birth weight (grams), underlying pathology, other pathologies, length of hospital stay (days), and outcome.

	**GA (weeks)**	**BW (g)**	**Underlying pathology**	**HS (d)**	**Outcome**
**Case 1**	34.7	1,640	Multiple (apple-peel) intestinal atresias	346	Discharge
**Case 2**	33.7	1,960	Vanishing gastroschisis^*^	242	Transfer^‡^
**Case 3**	30.4	1,700	OEIS	690	Discharge
**Case 4**	34.4	2,290	Vanishing gastroschisis	678	Transfer^‡^
**Case 5**	31.7	2,180	Berdon syndrome ^†^	600	Transfer^‡^
**Case 6**	33.8	2,350	Berdon syndrome ^†^	420	Transfer^‡^

GA (weeks) = gestational age in weeks; BW (g) = birth weight in grams; HS (d) = length of hospital stay in days; OEIS = omphalocele, cloaca exstrophy, imperforate anus, and spina bifida complex.

* Vanishing gastroschisis: a situation in which an abdominal wall defect closes in utero, cutting off the intestinal blood supply and causing major intestinal necrosis;

† Berdon syndrome: giant bladder, microcolon, and intestinal hypoperistalsis;

‡ Transferred to the ward.

**Table 2:** Age at insertion of the catheter (days), type of catheter, implantation technique, and length of stay (days).

	**Age at insertion of catheter (days)**	**Type of catheter** ^*^	**Implantation technique**	**Length of stay (days)**	**Catheter changes**
Case 1	158	Silicone	Venous cutdown	161	Zero
Case 2	38	PowerPicc (PICC Power Sinergy™, São Paulo)	Puncture	204	Zero
Case 3	261	Silicone	Puncture	116	Zero
Case 4	192	Silicone	Venous cutdown	507	One
Case 5	58	Groshong (Groshong™ Central Venous Catheter BD, São Paulo)	Venous cutdown	271	Zero
Case 6	85	PowerPicc	Puncture	67	Two

* All catheters were tunneled and all were double lumen catheters.

**Table 3:** Episodes of catheter-related infection and agents isolated from paired blood cultures (central and peripheral).

	**Central venous catheter-related infection**	**Agents isolated**		
		**Gram +**	**Gram** **-**	**Fungi**
Case 1	Present	*S. epidermidis*	Absent	Absent
		*S. hominis*		
Case 2	Absent	Absent	Absent	Absent
Case 3	Present	*S. epidermidis*	Absent	Absent
Case 4	Present	*S. epidermidis*	*E. coli*	*C. albicans*
			*E. faecium*	
			*K. aerogenes*	
Case 5	Present	*S. capitis*	*P. aeruginosa*	Absent
Case 6	Present	*S. aureus*	*E. coli*	*C. albicans*
			*E. faecalis*	
			*E. cloacae*	

The project was approved by the Pediatrics Department Ethics Committee and by the Research Project Analysis Committee at the Hospital das Clínicas da Faculdade de Medicina da Universidade de São Paulo (HC-FMUSP), under protocol No. 4.916.326.

## DISCUSSION

Children with short bowel syndrome have higher rates of bloodstream infections than children who need long-stay venous catheters but don’t have short bowel syndrome. Children with short bowel syndrome are subject to multiple risk factors for infection because, in addition to their underlying disease, which predisposes to extreme bacterial growth and involves intestinal dysmotility, they are also dependent on parenteral nutrition, are extremely young (less than 2 years old), and may develop malnutrition, increasing the risk of infections further still.^8^ Vascular access is important to enable survival of children with severe intestinal dysfunction.^10^

Late complications of vascular access include breakage, leaks, inadvertent traction, obstruction, and catheter-related bloodstream infections, while the most common complications are infections and thromboses.^11^

There were no immediate complications of catheter insertion in this case series. Additionally, no devices had to be removed because of obstructions, whether due to fibrin deposition or thrombosis.

Infection is the principal cause of long-dwell catheter loss. Infections can be caused by contamination at insertion or migration of microorganisms from the skin to the catheter, contamination by incorrect handling, or hematogenous contamination from remote sites.^12^ Risk factors for catheter-related infection include insertion technique and site, the type of catheter and number of lumens, duration of use, and type of infusion.^13^

Rates of infections associated with long term vascular accesses vary from 0.6 to 27%, depending on type of catheter, location, underlying disease, and catheter care. When there is fever, other etiologies of infection must be ruled out, making it obligatory to culture paired blood samples (peripheral and central). Staphylococcus are the most common causative agents of catheter-related bloodstream infections, followed by Gram-negative microorganisms and fungi.^12^^,^ 14 ^,^ 15 With tunneled catheters, more conservative treatment can be attempted for infections at the site of insertion, with the aim of salvaging the catheter.^14^ Removal of the catheter is mandatory if a culture from a systemic catheter-related infection is positive for *Staphylococcus aureus* or *Candida sp*.^15^

In the series described, 83.3% of the children had at least one catheter-related infection, distributed as follows: 83.3% with Gram-positive bacteria, 50% with Gram-negative bacteria, and 33.3% with fungi.

Colonization of intravascular catheters by microorganisms is a well-known phenomenon. Catheter lock is a technique that aims to degrade the biofilm to decontaminate the internal surface of the catheter, using concentrated doses of antibiotics, with or without heparin and fibrinolytics or ethanol. Biofilms are three-dimensional matrices consisting of platelets, plasma, fibronectin, and fibrinogen, which microorganisms can colonize, before detaching and reaching the bloodstream.^16^

Antimicrobial concentrations high enough to eradicate bacteria in biofilms are not achieved with systemic antibiotic treatment at recommended doses. Highly-concentrated antimicrobial solutions (antibiotic locks) administered into the venous catheter have therefore achieved better results. However, prophylaxis with antibiotics is subject to the potential disadvantage of development of bacterial resistance. Use of a vancomycin lock, for example, could provoke selection of *Enterococcus* resistant to this antibiotic.^1^

Ethanol is an antiseptic with bactericidal and fungicidal action against a wide range of Gram-positive and Gram-negative bacteria and fungi. The first reports of successful use of ethanol lock were in cancer patients and later in patients on total parenteral and prolonged nutrition.^17^^,^ 18

There are many advantages to using ethanol for prophylaxis. It is a substance with few side effects, good penetration of biofilms, and anticoagulant and fibrinolytic properties, that acts to lyse the cell wall, causing death of the microorganism, but without inducing antimicrobial resistance.^18^^,^ 19

The technique involves injecting 70% ethanol into the catheter lumen and allowing the solution to remain there for a certain amount of time, with the objective of preventing colonization or of sterilization of the lumen.^1^^,^ 17

Despite the potential beneficial effects of administration of ethanol lock, this case series only revealed a relative advantage, since all of the children were administered the ethanol lock daily for 4 hours, as recommended, and even so 83.3% of them had an infection. It should be mentioned that case 2 did not suffer a single infectious episode and the same catheter remained in place until transfer, which occurred 204 days after catheter insertion. Furthermore, there was only one *Staphylococcus aureus* infection episode, in a different child, even though this is a highly prevalent agent in central venous catheter-related bloodstream infections, according to reports in the literature.^15^ Catheters were changed in three children (50% of cases), as recommended in the literature,^15^ because of fungal infection (*Candida albicans)* in two children and because of the *Staphylococcus aureus* infection in a third child. Another point in favor of the ethanol lock is the 182.5 day median catheter dwell duration, ranging from 67 to 507 days.

Potential toxic effects related to the ethanol lock include effects on the central nervous system (lethargy, anomalous movements, and convulsions), cardiac arrhythmias, and local venous irritation.^20^ However, there were no collateral effects during administration of the ethanol lock in any of the six children studied. Use of ethanol lock can be linked to structural changes to the molecules of the polymers in the catheters, especially when made from polyurethane, increasing the risk of obstruction and loss of catheter integrity, in addition to the systemic toxicity described above.^21^ These data were the reason for exclusive use of silicone catheters in patients administered ethanol lock.

In conclusion, the ethanol lock technique did not cause side effects and had relative efficacy for prevention of venous catheter-related infections. Although the majority of studies with ethanol lock in silicone catheters in children also do not report adverse events, these results should be interpreted with caution since they are retrospective and the sample size is small.^21^ Studies with larger numbers of cases are needed to demonstrate the true efficacy of ethanol lock for prevention of bloodstream infections related to use of central venous catheters.

Since studies with ethanol lock in children with severe intestinal insufficiency are scarce, data have been extrapolated from meta-analyses including adults and some pediatric patients without intestinal dysfunction, in order to extend the discussion.

A meta-analysis including 2,575 patients and 3,375 catheters from seven controlled and randomized studies showed that ethanol lock was effective for reduction of bloodstream infections in adult patients on hemodialysis with tunneled central venous catheters.^22^ Still in relation to adult patients on hemodialysis, a meta-analysis of data from 7,020 patients showed that both ethanol and antibiotic locks had efficacy for prevention of infections.^23^

Another meta-analysis, from 2020, including adult patients on parenteral nutrition at home concluded that taurolidine was the most effective lock solution for prevention of central catheter-related infections.^24^

Finally, despite certain limitations, a meta-analysis including adult patients given ethanol lock showed a positive effect for reduction of central catheter-related infections when compared to use of heparin in isolation, concluding that prophylaxis with ethanol is a potential candidate for prevention of infections in these patients.^25^

Therefore, the results of the ethanol lock technique in pediatric patients with short bowel syndrome could be promising. However, data are insufficient to support formal recommendation of the practice, since there are very few publications and the majority involve small numbers of patients.^8^
