# Supplementary material for: Ethanol lock prophylaxis in long-stay central venous catheters in children with severe intestinal dysfunction: a report of six cases
Source: J Vasc Bras. 2022 Jul 6;21:e20210221. doi: 10.1590/1677-5449.202102211 (PMC9296061; doi:10.1590/1677-5449.202102211)
Supplement: Translation

# Translation: Profilaxia com *lock* de etanol em cateter venoso central de longa permanência em crianças com disfunção intestinal grave: relato de seis casos

**DOI:** 10.1590/1677-5449.202102212

**Authors:** Mário Cícero Falcão, Gabriela Ibrahim Martins de Castro, Juliana Valeria de Souza Framil, Juliana Zoboli Del Bigio, Ana Cristina Aoun Tannuri

**Affiliations:** 1 Universidade de São Paulo – USP, Instituto da Criança e do Adolescente, Faculdade de Medicina, Hospital das Clínicas, São Paulo, SP, Brasil.

**Keywords:** síndrome do intestino curto, nutrição parenteral total, infecções relacionadas a cateter, etanol

## Abstract

O objetivo deste estudo foi relatar o uso de *lock* de etanol na profilaxia infecciosa de cateteres venosos de longa permanência em recém-nascidos com disfunção intestinal grave e dependentes de nutrição parenteral total e prolongada, internados em um Centro de Terapia Intensiva Neonatal (nível terciário) entre 2015 e 2020. Das 914 admissões, seis (0,65%) recém-nascidos preencheram os critérios de inclusão. A mediana da idade da passagem do cateter foi de 121,5 dias, sendo dois cateteres PowerPicc (PICC Power Sinergy™, São Paulo), um cateter Groshong (Groshong™ Central Venous Catheter BD, São Paulo) e três cateteres de silicone, todos tunelizados. O tempo de permanência apresentou mediana de 182,5 dias. Cinco pacientes apresentaram pelo menos um episódio de infecção associada ao cateter venoso central, sendo isolados agentes Gram-positivos, negativos e fungos. A mediana de dias de internação foi de 555, e a mortalidade, 33,3%. O *lock* de etanol não apresentou efeitos colaterais e foi relativamente eficaz na prevenção de infecções relacionadas ao cateter venoso central.

## INTRODUÇÃO

A perda de funções intestinais pode ser resultado de ausência de intestino, de distúrbios de absorção, de motilidade ou de uma combinação dessas situações. Um exemplo clássico dessa associação é a síndrome do intestino curto, em que falta intestino, principalmente delgado, para permitir digestão e absorção adequadas de nutrientes para a manutenção da vida^1^^,^ 2.

A causa mais comum da síndrome do intestino curto resultante da ressecção cirúrgica em recém-nascidos é a enterocolite necrosante (35 a 50% dos casos). Outras situações são defeitos da parede abdominal (gastrosquises e onfaloceles), vícios de rotação, volvos e atresias intestinais múltiplas^3^^,^ 4. Apesar das causas, a abordagem da disfunção intestinal é a mesma e inclui promover adaptação e recuperação intestinal para permitir sua autonomia, ou seja, independência de nutrição parenteral^5^.

Complicações sépticas associadas a infecções do cateter venoso central são as principais causas de mortalidade, com taxas variando entre 20 e 40%, na dependência da prevenção dessas complicações^6^.

A infecção primária de corrente sanguínea é considerada relacionada ao cateter venoso central quando se documenta que há maior inócuo do patógeno no cateter do que no sangue periférico, seja pela análise quantitativa de cultura da ponta do cateter (identificação do mesmo patógeno do sangue periférico, com mais de 15 UFC/placa em cultura semiquantitativa de ponta de cateter ou mais de 100 UFC/mL em cultura quantitativa), pela diferença de concentração entre hemoculturas centrais e periféricas (crescimento pelo menos três vezes maior em hemocultura quantitativa de sangue de cateter venoso central do que de sangue periférico) ou pela diferença no tempo de positividade entre as hemoculturas centrais e periféricas (crescimento mais rápido em hemocultura de cateter venoso central, sendo a diferença de positividade maior que 120 minutos)^7^.

Assim, esforços vêm sendo empregados no sentido de se diminuir o risco de infecções relacionadas ao cateter venoso central, como reforço à prática correta de higiene de mãos, cuidados durante a inserção dos cateteres, adoção de pacote de medidas (*bundles*) que abordam cuidados na manutenção dos cateteres, como maior vigilância dos curativos e retirada precoce dos dispositivos, quando possível, e cuidados relacionados à manipulação dos cateteres, especialmente durante o banho, a coleta de exames e a realização de medicações^7^.

Além das medidas básicas, pode-se lançar mão do uso de substâncias para evitar a formação de biofilme no cateter, técnica conhecida como *lock* profilático^8^. Entre os métodos para se evitar formação de biofilmes, destacam-se os bloqueios com antibióticos, fibrinolíticos e etanol^1^. Uma revisão sistemática, publicada em 2017, incluindo pacientes pediátricos e adultos com câncer mostrou que a técnica de bloqueio com antimicrobianos pode ser um adjuvante para a prevenção de infecção de corrente sanguínea em pacientes com cateter venoso central. No entanto, evidências de maior qualidade são necessárias para recomendações específicas^9^.

## DESCRIÇÃO DOS CASOS

Os critérios de inclusão dos casos clínicos foram os seguintes: presença de insuficiência intestinal grave, jejum prolongado, nutrição parenteral total infundida por cateter venoso central de silicone implantado por técnica de tunelização e realização de *lock* profilático com etanol por 4 horas. Entre 2015 e 2020, foram admitidos 914 recém-nascidos na unidade; destes, seis preencheram os critérios listados acima.

As Tabelas 1, 2 e 3 resumem a descrição dos casos, salientando-se que 66,6% apresentavam síndrome do intestino curto; a mediana do tempo de permanência do cateter foi de 182,5 dias. Não foram observados efeitos colaterais com o emprego do *lock* profilático com etanol, e 83,3% dos pacientes apresentaram pelo menos um episódio de infecção relacionada ao cateter venoso central, sendo isolados agentes Gram-positivos, negativos e fungos. A mediana de internação foi de 555 dias, e a mortalidade, 33,3%, ressaltando que nenhum óbito ocorreu na unidade. A causa dos três óbitos foi sepse com falência de múltiplos órgãos.

**Tabela 1:** Idade gestacional (semanas), peso de nascimento (gramas), patologia de base, outras patologias, tempo de internação (dias) e desfecho.

	IG (sem)	PN (g)	Patologia de base	TI (d)	Desfecho
**Caso 1**	34,7	1.640	Múltiplas atresias intestinais (*apple-peel*)	346	Alta
**Caso 2**	33,7	1.960	*Vanishing* gastrosquise^*^	242	Transferência^‡^
**Caso 3**	30,4	1.700	OIES	690	Alta
**Caso 4**	34,4	2.290	*Vanishing* gastrosquise	678	Transferência^‡^
**Caso 5**	31,7	2.180	Síndrome de Berdon^†^	600	Transferência^‡^
**Caso 6**	33,8	2.350	Síndrome de Berdon^†^	420	Transferência^‡^

IG (sem) = idade gestacional em semanas; PN (g) = peso de nascimento em gramas; TI (d) = tempo de internação em dias; OIES = associação de onfalocele, ânus imperfurado, extrofia de cloaca e espinha bífida.

* *Vanishing* gastrosquise: situação em que o defeito da parede abdominal se fecha no período intrauterino, causa interrupção de suprimento sanguíneo intestinal e grande necrose intestinal;

† Síndrome de Berdon: megabexiga, microcólon e hipoperistaltismo intestinal;

‡ Transferência para enfermaria.

**Tabela 2:** Idade na implantação do cateter (dias), tipo de cateter, técnica de implantação e tempo de permanência (dias).

	**Idade na implantação do cateter (dias)**	**Tipo de cateter** *	**Técnica de implantação**	**Tempo de permanência (dias)**	**Troca de cateter**
Caso 1	158	Silicone	Venodissecção	161	Não
Caso 2	38	PowerPicc (PICC Power Sinergy™, São Paulo)	Punção	204	Não
Caso 3	261	Silicone	Punção	116	Não
Caso 4	192	Silicone	Venodissecção	507	Uma troca
Caso 5	58	Groshong (Groshong™ Central Venous Catheter BD, São Paulo)	Venodissecção	271	Não
Caso 6	85	PowerPicc	Punção	67	Duas trocas

* Todos os cateteres foram implantados com técnica de tunelização, e todos os cateteres eram duplo lúmen.

**Tabela 3:** Episódios de infecção relacionada ao cateter e agentes isolados de hemoculturas pareadas (centrais e periféricas).

	Infecção relacionada ao cateter venoso central	Agentes isolados		
		Gram +	Gram -	Fungos
Caso 1	Presente	*S. epidermidis*	Ausentes	Ausentes
		*S. hominis*		
Caso 2	Ausente	Ausentes	Ausentes	Ausentes
Caso 3	Presente	*S. epidermidis*	Ausentes	Ausentes
Caso 4	Presente	*S. epidermidis*	*E. coli*	*C. albicans*
			*E. faecium*	
			*K. aerogenes*	
Caso 5	Presente	*S. capitis*	*P. aeruginosa*	Ausentes
Caso 6	Presente	*S. aureus*	*E. coli*	*C. albicans*
			*E. faecalis*	
			*E. cloacae*	

O projeto foi aprovado pelo Comitê de Ética do Departamento de Pediatria e pelo Comitê de Análise de Projetos de Pesquisa (CAPPesq) do Hospital das Clínicas da Faculdade de Medicina da Universidade de São Paulo (HC-FMUSP), protocolo n.º 4.916.326.

## DISCUSSÃO

As crianças com síndrome do intestino curto apresentam altas taxas de infecções de corrente sanguínea quando comparadas a crianças que necessitam do uso de cateteres venosos de longa permanência sem intestino curto. Essas crianças com síndrome do intestino curto apresentam risco multifatorial para desenvolver quadros infecciosos, pois, além da doença de base, que cursa com supercrescimento bacteriano e dismotilidade intestinal, esses pacientes são dependentes de nutrição parenteral, encontram-se no extremo da idade (abaixo de 2 anos) e podem também evoluir com desnutrição, aumentando o risco de infecções^8^. Assim, o acesso vascular é importante para possibilitar a sobrevivência de crianças com disfunção intestinal grave^10^.

As complicações tardias dos acessos vasculares consistem em quebra, extravasamentos, tração inadvertida, obstrução e infecções de corrente sanguínea relacionadas ao cateter, ressaltando que as complicações mais comuns são infecções e tromboses^11^.

Nessa série de casos, não houve nenhuma complicação imediata da passagem do cateter. Também não houve nenhuma perda do dispositivo por obstrução, seja por deposição de fibrina ou trombose.

A infecção é a principal causa de perda do cateter de longa permanência. Essa situação pode ocorrer por contaminação da inserção ou migração de microrganismos da pele para o cateter, contaminação por manipulação inadequada ou contaminação por via hematogênica, proveniente de outros sítios^12^. São considerados fatores de risco para infecção relacionada ao cateter a técnica e o sítio de inserção, o tipo de cateter e o número de lúmens, o tempo de uso e o tipo de infusão^13^.

A taxa de infecções associadas a acessos vasculares em longo prazo varia de 0,6 a 27%, dependendo do tipo de cateter, da localização, da doença de base e dos cuidados dispensados ao cateter. No caso de febre, devem-se excluir outras etiologias de infecção, sendo obrigatória a coleta de hemoculturas pareadas (periférica e central). Os agentes mais comuns de infecção de corrente sanguínea relacionada ao cateter são os estafilococos, seguidos de microrganismos Gram-negativos e fungos^12^^,^ 14 ^,^ 15. Em cateteres tunelizados, ante uma infecção no local de inserção, um tratamento mais conservador pode ser instituído na tentativa de se preservar o cateter^14^. No caso de infecção sistêmica relacionada ao cateter com culturas positivas para *Staphylococcus aureus* ou *Candida sp*, a retirada do cateter é mandatória^15^.

Na série descrita, 83,3% das crianças apresentaram pelo menos uma infecção relacionada ao cateter, assim distribuídas: 83,3% com Gram-positivos, 50% com Gram-negativos e 33,3% com fungos.

É bem conhecida a ocorrência de colonização de cateteres intravasculares por microrganismos. O bloqueio é uma técnica que visa degradar o biofilme com o intuito de descontaminar a superfície interna do cateter, utilizando doses concentradas de antibióticos, com ou sem heparina e fibrinolíticos ou etanol. Os biofilmes são matrizes tridimensionais constituídas de plaquetas, plasma, fibronectina e fibrinogênio, em que microrganismos podem colonizar, desprender e atingir a corrente sanguínea^16^.

As concentrações antimicrobianas suficientes para erradicar bactérias em biofilmes não são alcançadas pela terapia sistêmica em doses recomendadas dos antibióticos. Assim, soluções antimicrobianas altamente concentradas (*lock* de antibióticos), administradas no cateter venoso, têm mostrado melhores resultados. No caso de profilaxia com antibiótico, no entanto, existe uma desvantagem: potencial risco para desenvolver resistência bacteriana. Um bloqueio com vancomicina, por exemplo, pode provocar seleção de *Enterococcus* resistentes ao antibiótico^1^.

O etanol é um antisséptico que mostra atividade bactericida e fungicida contra uma ampla gama de bactérias Gram-positivas, negativas e fungos. Os primeiros relatos bem-sucedidos sobre o uso do *lock* de etanol foram em pacientes oncológicos e, posteriormente, em pacientes recebendo nutrição parenteral total e prolongada^17^^,^ 18.

O uso de etanol como profilaxia tem muitas vantagens. Trata-se de uma substância com poucos efeitos colaterais, boa penetração nos biofilmes e propriedades anticoagulantes e fibrinolíticas, atuando na lise da parede celular, com consequente morte do microrganismo, sem promover resistência antimicrobiana^18^^,^ 19.

A técnica envolve injetar etanol 70% no lúmen do cateter e permitir que a solução permaneça por certo período de tempo, com o objetivo de prevenir a colonização ou promover a esterilização do lúmen^1^^,^ 17.

Apesar dos potenciais efeitos benéficos da aplicação do bloqueio com etanol, esta série de casos mostrou somente relativa vantagem, pois todas as crianças receberam *lock* de etanol diário por 4 horas, conforme recomendado, e, mesmo assim, 83,3% apresentaram infecção sanguínea relacionada ao cateter venoso central. Vale ressaltar que o caso 2 não apresentou nenhum episódio infeccioso, e o mesmo cateter permaneceu até a sua transferência, que ocorreu após 204 dias da passagem do cateter, além da ocorrência de somente um episódio infeccioso, em outra criança, por *Staphylococcus aureus*, que é um agente bastante prevalente, conforme relatos da literatura, em infecções sanguíneas relacionadas ao cateter venoso central^15^. Conforme recomenda a literatura^15^, a troca de cateter ocorreu em três crianças (50% dos casos), ante a presença de infecção fúngica (*Candida albicans)* em duas crianças e de infecção por *Staphylococcus aureus* um uma. Outro ponto a favor do *lock* de etanol é o tempo de permanência dos cateteres de 182,5 dias (mediana), variando de 67 a 507 dias.

Potenciais efeitos tóxicos relacionados ao *lock* de etanol incluem ações no sistema nervoso central (letargia, movimentos anômalos e convulsões), arritmias cardíacas e irritação venosa local^20^. No entanto, nenhum efeito colateral ocorreu durante a aplicação do bloqueio de etanol nas seis crianças estudadas. O uso do bloqueio de etanol pode-se associar a alterações estruturais das moléculas dos polímeros dos cateteres, especialmente quando confeccionados com poliuretano, resultando em aumento do risco de obstrução e quebra na integridade do cateter, além da toxicidade sistêmica descrita acima^21^. Essas informações justificam o uso exclusivo de cateteres de silicone ante a utilização de bloqueio de etanol.

Concluindo-se, a técnica de bloqueio com etanol não apresentou efeitos colaterais e mostrou relativa eficácia na prevenção de infecções relacionadas ao cateter venoso. Apesar de a maioria dos estudos com bloqueio de etanol em cateteres de silicone em crianças não relatar eventos adversos, a interpretação desses resultados merece cautela, pois são retrospectivos e com pequeno tamanho amostral^21^. Estudos com maior número de casos precisam ser realizados para demonstrar a real eficácia desse bloqueio na prevenção de infecção sanguínea relacionada ao cateter venoso central.

Como os estudos com bloqueio de etanol em crianças com insuficiência intestinal grave são escassos, alguns dados foram extrapolados de metanálises incluindo pacientes adultos e alguns pediátricos sem disfunção intestinal para enriquecer a discussão.

Uma metanálise incluindo 2.575 pacientes e 3.375 cateteres de sete estudos controlados e randomizados mostrou que o *lock* de etanol foi efetivo na redução de infecção de corrente sanguínea em pacientes adultos em hemodiálise com cateter venoso central tunelizado^22^. Ainda em relação a pacientes adultos em hemodiálise, uma metanálise reunindo 7.020 pacientes mostrou eficácia tanto do *lock* de etanol quanto de antibióticos na prevenção de infecções^23^.

Outra metanálise, de 2020, incluindo pacientes adultos recebendo nutrição parenteral domiciliar concluiu que a taurolidina se mostrou mais efetiva na prevenção de infecções relacionadas ao cateter central^24^.

Por fim, uma metanálise incluindo pacientes adultos que realizaram bloqueio com etanol mostrou, apesar das limitações, um efeito positivo na redução de infecções relacionadas ao cateter central, quando comparado com o uso isolado de heparina, concluindo que a profilaxia com etanol é uma potencial candidata para prevenção de infecções nesses pacientes^25^.

Assim, a técnica de *lock* de etanol em pacientes pediátricos com síndrome do intestino curto pode ter resultados promissores. Entretanto, faltam dados para a recomendação formal da prática, uma vez que há poucas publicações e a maioria envolve um número pequeno de pacientes^8^.
